# A personalised nutrition intervention for adolescent depression: a mixed-methods feasibility pilot study

**DOI:** 10.1017/S0007114524001338

**Published:** 2026-03-14

**Authors:** Susan C. Campisi, Megan Liang, Samantha J. Anthony, Elizabeth Dettmer, Daphne J. Korczak

**Affiliations:** 1 Neuroscience and Mental Health, The Hospital for Sick Children, Toronto, Canada; 2 Nutrition and Dietetics Program, Clinical Public Health Division, Dalla Lana School of Public Health, University of Toronto, Toronto, Canada; 3 Child Health Evaluative Sciences Program, The Hospital for Sick Children, Toronto, Canada; 4 Factor-Inwentash Faculty of Social Work, University of Toronto, Toronto, Canada; 5 Department of Psychology and the Healthy Living Clinic, The Hospital for Sick Children, Toronto, Canada; 6 Department of Paediatrics, Temerty Faculty of Medicine, University of Toronto, Toronto, Canada; 7 Department of Psychiatry, Temerty Faculty of Medicine, University of Toronto, Toronto, Canada

**Keywords:** Dietary intervention, Pilot, Adolescents, Depression

## Abstract

Randomised controlled trials have demonstrated the benefit of diet modification to improve diet quality in the treatment of adult major depressive disorder (MDD). However, research examining nutritional interventions for adolescents with MDD is sparse. This pilot study examined the feasibility of a personalised nutrition intervention for adolescents with MDD. Ten adolescents with MDD and their parents recruited from a tertiary care setting participated in an 8-week, single-arm mixed-methods study. Feasibility was assessed using five criteria (demand, acceptability, implementation, adaptation and limited efficacy testing) alongside qualitative interviews. The intervention involved four bi-weekly virtual nutrition counselling sessions with a stepped approach to dietary change, menu planning, grocery delivery and educational eHealth messages. Study participants sought positive changes in diet, health and lifestyle for adolescents and family-wide benefits. Recruitment challenges included concerns about managing mood fluctuations, anticipated dietary restrictions and the potential time and effort required for diet adherence. Feedback based on interviews emphasised moderate to high acceptability, satisfaction with menu planning and counselling and recognition of the benefits of trying new foods and sustaining positive dietary changes beyond the study. Improvements in depression symptoms (Cohen’s *d* = 0·36, 95 % CI (–0·24, 3·36)), parent food modeling (Cohen’s *d* = 0·24, 95 % CI (–0·43, 1·16) and the family food environment (Cohen’s *d* = 0·61, 95 % CI (–0·04, 2·61)) were observed. This nutrition intervention was feasible for adolescents with MDD and was acceptable to both parents and depressed adolescents. These preliminary data suggest that further examination of the intervention and its potential benefits on depression symptoms and family food dynamics are warranted.

Depression is a major contributor to the global disease burden^(1,2)^. With an estimated worldwide prevalence of 31·53 million cases (95 % CI: 27·2, 36·5), depression impacts physical and mental well-being, leading to socio-economic consequences, reduced quality of life and an increased burden on healthcare systems^(3)^. As approximately 70 % of adult mental health disorders, including depression, emerge in childhood or early adolescence^(4)^, the public health impact of adolescent-onset depression may be even greater, as depression is both an independent source of impairment as well as a risk factor for other chronic health conditions later in life (e.g. obesity, type two diabetes mellitus, ischaemic heart disease)^(5–7)^. Despite advances in treatments, many children and adolescents do not respond to first-line depression treatment(s), and treatment resistance occurs in 30–40 % of cases^(8)^. As highlighted by the 2018 Lancet Commission on Psychiatry and the 2021 State of the World’s Children Report, there is an urgent need for innovative therapeutic interventions for children and adolescents with depression^(9,10)^.

Diet modification offers promise in improving both mental and physical health. Lifestyle factors such as healthy eating have been shown to play a pivotal role in the treatment and prevention of numerous non-communicable chronic diseases, and their impact on mental health is being increasingly recognised^(11,12)^. Some of the physiological pathways hypothesised to be affected by diet in relation to depression include oxidative stress^(13)^, inflammation^(14,15)^, mitochondrial function^(16)^, the gut microbiome (via the gut–brain axis)^(17)^ and disruptions of tryptophan–kynurenine metabolism^(18)^. Research in adults has demonstrated an association between greater adherence to healthy diets, such as the Mediterranean diet and reduced depressive symptoms^(19–22)^. In addition, greater adherence to healthy dietary patterns is associated with lower depressive symptoms among both clinical and community-based samples of adolescents^(21,23,24)^ and adults^(21,25,26)^. Moreover, among adolescents with major depressive disorder (MDD), lower dietary quality is associated with higher depressive symptoms^(27)^.

As adolescence is a developmental period during which dietary habits become entrenched^(28)^, and as unhealthy dietary patterns track between adolescence and adulthood^(29,30)^, the period of adolescence represents a potential window of opportunity for intervention to improve eating behaviours lifelong. However, factors that influence adolescent food choices are complex and are also nested in family factors, such that interventions targeting adolescent dietary patterns must also be multifaceted. Thus, nutrition interventions should encompass the adolescent food environment and include elements that address nutrition literacy^(31)^, family eating habits and practices^(32,33)^, peer influences^(34)^, school environments^(35)^ as well as broader cultural and societal norms^(36,37)^.

The overarching objective of this study was to assess the feasibility of a novel personalised nutrition intervention for adolescents with MDD using a mixed-methods study design. The secondary objective was to gather preliminary data to inform a larger, appropriately powered study to test the personalised nutrition intervention’s effectiveness in improving dietary intake and depressive symptoms.

## Methods

### Study design

This 8-week open, single-arm study assessed the feasibility of implementing a personalised nutrition intervention, based on the principles of a Mediterranean diet, among adolescents with MDD. The study combined quantitative and qualitative data to more completely evaluate the intervention and the study protocol^(38)^.

This study was approved by The Hospital for Sick Children Research Ethics Board #1000079300 on March 20, 2022. Recruitment and intervention delivery occurred from May 2022 to July 2023. All adolescents and their guardians provided written informed consent. The study was registered at the US National Institutes of Health (ClinicalTrial.gov) #NCT06175052.

### Participants

The study recruited ten adolescent–parent dyads. Adolescents were recruited from the Children’s Integrated Mood and Body (CLIMB) depression program at The Hospital for Sick Children (SickKids), a tertiary care children’s hospital in Toronto, Canada. The CLIMB programme receives referrals from primary care clinicians in the community via the centralised intake programme in the outpatient psychiatry department at SickKids. Adolescents referred to the CLIMB programme undergo semi-structured diagnostic interviews by a trained staff member using the Kiddie Schedule for Affective Disorders and Schizophrenia (K-SADS)^(39)^, and psychiatric diagnoses, including MDD, are confirmed following a review with a child and adolescent psychiatrist. Dyads who expressed interest in participating in the current study provided their consent and completed screening questionnaires to determine their eligibility. Participants received usual clinical care during the study. Details regarding the CLIMB programme have been published elsewhere^(40)^.

Study inclusion criteria were the following: adolescent age 11–17 years; diagnosis of MDD as defined by the Diagnostic and Statistical Manual for Mental Disorders 5th edition (DSM-5)^(41)^; access to the internet and a computer or smartphone and a parent willing to participate. Adolescents were excluded if they already adhered to a high-quality diet (i.e. a score ≥ 8 on the KIDMED^(42)^); met the criteria for an eating disorder based on the K-SADS^(39)^ semi-structured interview; were currently participating in other dietary programs or studies or were actively attempting to increase or decrease body weight; had a chronic medical condition not including MDD or had an unstable psychiatric disorder (e.g. manic symptoms, active suicidal ideation). Parents and/or legal guardians of adolescents were eligible to participate if they lived with the adolescent at least half of the time and had access to the internet and a computer or smartphone.

### Sample size

This study, designed to assess the feasibility of a dietary intervention for adolescents with depression, employed a sample of ten participants. Other feasibility studies conducted among adolescents with depression use similar sample sizes ranging from 10 to 13 participants^(43–45)^. This feasibility stage focussed on gathering initial data regarding participant recruitment, intervention adherence and data collection procedures. A sample size of ten enabled evaluation of these aspects and will inform the design of a future pilot study with a more robust sample size capable of yielding statistically significant results.

### Study procedure

Following enrollment, adolescents and parents completed independent pre-intervention interviews. Adolescents completed baseline questionnaires about depression symptoms, dietary intake, nutrition knowledge and attitudes, parental food modelling as well as perceived intervention acceptability and feasibility. Parents completed baseline questionnaires about the family food environment and perceived intervention acceptability and feasibility.

**Fig. 1.**
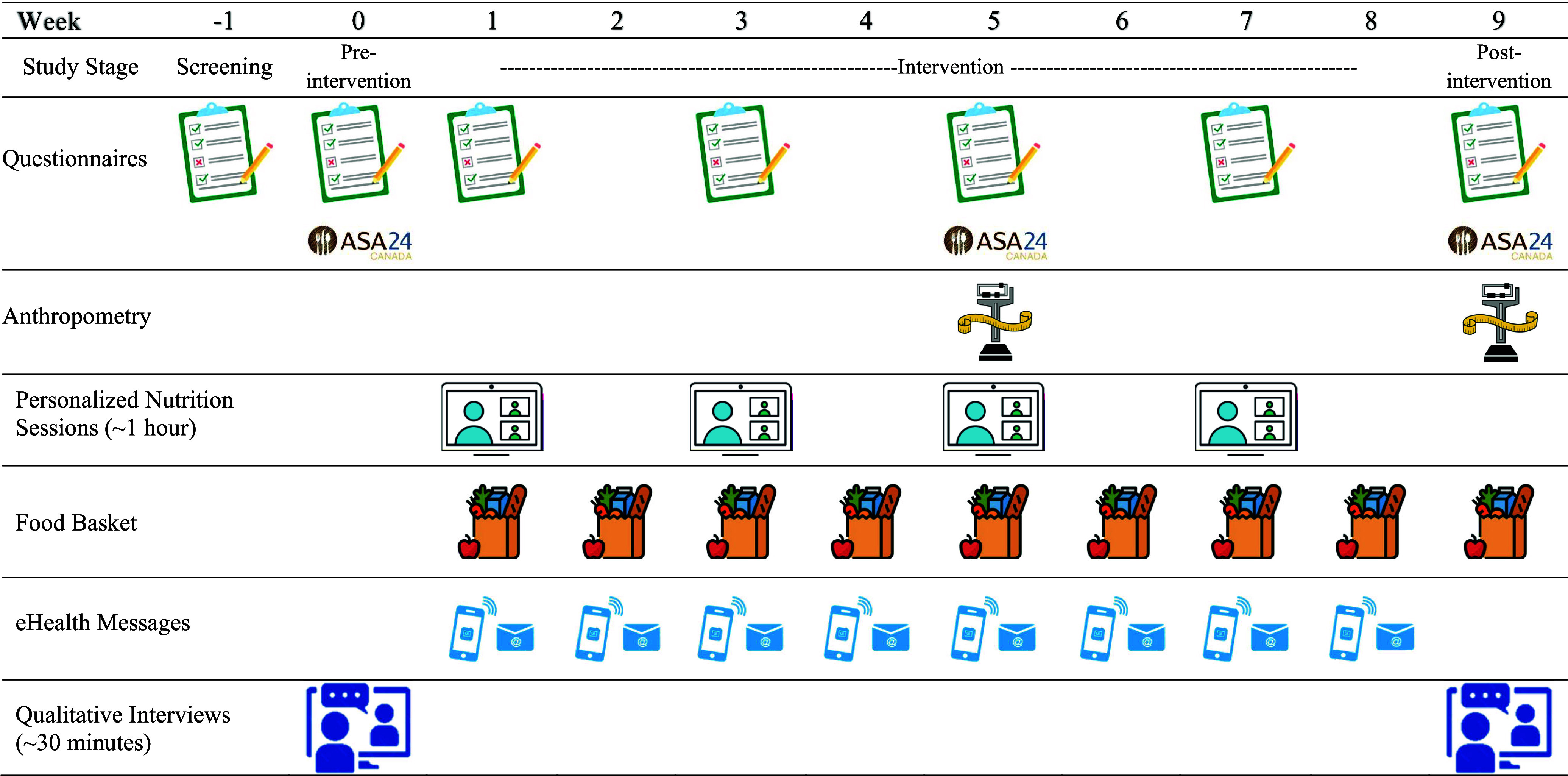
Schedule of study assessments and activities.

Following the assessment, participants began the 8-week personalised nutrition intervention. Nutrition counselling session feedback was obtained via a questionnaire after each session. In Week 5 and Week 9 (1 week post-intervention), adolescent self-report and parent-report measures were repeated. Adolescents and parents completed independent post-intervention interviews following the completion of the intervention to elicit further feedback about their experiences. The study procedure is further detailed in Fig. 1.

### Personalised nutrition intervention

The personalised nutrition intervention included four virtual nutrition counselling sessions, provided every 2 weeks for 8 weeks. The nutrition counselling sessions followed a structured five-step approach known as the 5A’s (Assess, Advise, Agree, Assist and Arrange)^(46)^. At each nutrition counselling session, adolescent–parent dyads and the nutrition counsellor collaboratively set one nutrition goal (i.e. eating two or more servings of fruit per day) and one eating behaviour goal (i.e. eating breakfast regularly). A 1-week personalised menu plan was then co-created based on the principles of the Mediterranean diet (i.e. higher intake of fruit, vegetables, grains, legumes and lean protein and lower intake of red meats and processed foods) for use over the following 2 weeks, using ‘That Clean Life’ menu planning software^(47)^. Participants also received weekly grocery deliveries containing food items from the menu plan and weekly eHealth messages by email. The eHealth messages were tailored to reinforce the principles of a high-quality diet. An additional grocery delivery was sent to each dyad after completing the post-intervention assessments.

### Quantitative measures

Quantitative measures were administered using REDCap^(48)^, a secure web-based platform for data collection, with the exception of the 24-h dietary record, which was accessed via the secure website link^(49)^.

#### Adolescent depression

Depression symptoms were assessed using the Centre for Epidemiological Studies Depression Scale for Children (CES-DC), a validated twenty-item self-report measure^(50)^. Participants rated their symptoms on a four-point Likert scale, with higher scores indicating more severe depression symptoms and a maximum total score of 60.

#### Dietary assessment

Adolescents’ adherence to the personalised nutrition intervention was assessed using the KIDMED questionnaire, a sixteen-item self- or interviewer-administered measure^(42)^. Items related to lower adherence are assigned a value of –1 (4 items), and those related to higher adherence are assigned a value of +1 (12 items). Scores range from 0 to 12. A total score of ≤ 3 indicates poor adherence; a score of 4–7 indicates moderate adherence and a score of ≥ 8 indicates high adherence.

The intended method for assessing diet quality was the Healthy Eating Index-2015 (HEI-2015).^(51)^ Two 24-hour dietary recalls at each time point were obtained using the National Cancer Institute’s Automated Self-Administered 24-hour (ASA-24) Dietary Assessment, a validated instrument that employs the Automated Multiple-Pass Method^(49)^. The ASA24 instrument has been shown to be a valid measure of dietary quality that is sensitive to change in studies of short-term (4–8 weeks) dietary interventions in adolescents and adults.^(52–56)^ The HEI-2015 is a scoring system based on density rather than quantity, with values ranging from 0 to 100. Total scores over 80 are categorised as good quality diets, those with scores 51–79 as needing improvement and those with scores ≤ 50 as being of poor quality. The HEI-2015 includes thirteen subgroups categorised into adequacy components and moderation components. Adequacy components are foods and nutrients that are encouraged, and higher scores reflect higher intakes. Moderation components are reverse-scored since they include foods and nutrients with recommended limits. Higher moderation scores reflect lower intakes since lower intakes better align with recommendations.

#### Anthropometry

Pre-intervention BMI data were obtained from standardised assessments during clinic visits, as detailed elsewhere^(40)^. To evaluate the feasibility of including body weight data within the study protocol, participants were instructed to weigh themselves while wearing light clothing with a parent present, using their home scales. Self-reported weights were measured in duplicate and submitted electronically via REDCap.

#### Nutrition attitudes and knowledge

We assessed nutrition attitudes and knowledge using a Nutrition Attitudes and Knowledge (NAK) questionnaire, designed to evaluate children’s understanding of the 2019 Canada Food Guide^(57)^. The questionnaire consists of four five-point Likert scale questions to measure positive attitudes about nutrition, as well as twenty multiple-choice and true/false questions related to Canada Food Guide food groups (drinks, whole grain foods, vegetables and fruit and protein foods).

#### Parent food modelling

Parental food modelling was evaluated with five items adapted from Cullen’s scale, each rated on a four-point Likert scale from ‘Never’ to ‘Always,’ which has been used to predict diet outcomes in adolescent samples^(58)^.

#### Family food environment

Parents used the Family and Nutrition and Physical Activity questionnaire, a twenty-item tool on a four-point Likert scale, to assess their family’s food environment and practices. Higher scores indicate healthier home food environments^(59)^.

#### Acceptability and feasibility of intervention measures (AIM and FIM)^(60)^

The AIM and FIM are self-reported four-item measures that are scored using a five-point Likert scale ranging from ‘completely disagree’ (1) to ‘completely agree’ (5) with higher scores indicating greater acceptability or feasibility. While there are no established cut-off scores for interpreting AIM and FIM data, most research evaluates the intervention’s reception by examining mean scores^(61)^. The maximum score achievable for each measure is 20. Higher mean scores for each item (closer to 5) indicate greater acceptability and feasibility.

Satisfaction with the menu planning and nutrition counselling was rated by parents and adolescents independently following each counselling session using a five-point Likert scale ranging from ‘very dissatisfied’ (1) to ‘very satisfied’ (5).

### Qualitative interviews

Semi-structured interviews were conducted using an interview guide and recorded virtually by members of the research team. The pre-intervention interview probed reasons for study participation and potential concerns, perceptions of the personalised nutrition intervention, participants’ expectations of the study and motivation for enrollment. The post-intervention interview asked participants about their perspectives of the intervention as a whole and its individual components, including the nutrition counselling sessions, grocery delivery and eHealth messages. Participants were also asked about the factors that either hindered or facilitated their adherence to the personalised nutrition intervention.

### Feasibility measures

Feasibility was evaluated based on the following key focus areas as delineated by Bowen *et al.* ^(62)^ – demand, acceptability, implementation, adaptation and limited efficacy testing. Qualitative interviews with youth and parents were integrated to enrich quantitative data for a more robust overall feasibility evaluation^(63)^.

*1. Demand,* the extent to which the intervention would be accessed by adolescents with MDD, was measured quantitatively with the recruited rate. The recruitment rate was defined as the number of dyads enrolled in the study divided by the number of dyads approached to participate in the study. Demand was also assessed through pre-intervention qualitative interviews.

*2. Acceptability,* the extent to which the intervention was judged to be suitable to dyads, and fit with daily life activities, was assessed using the AIM. Acceptability was also assessed using post-intervention qualitative interviews. Satisfaction with the menu planning and nutrition counselling sessions was assessed using the quantitative satisfaction scores, and through post-intervention qualitative interviews.

*3. Implementation*, the degree to which the intervention and questionnaires can be delivered as proposed, was assessed using the FIM and by intervention adherence, participant attrition and the completion rate of the quantitative measures. Adolescent dietary adherence was assessed using the KIDMED questionnaire. The attrition rate was defined as the number of dyads who started the intervention divided by the number of dyads who completed the study, expressed as a percentage. The degree of missingness examined the proportion of missing data (i.e. potentially indicative of questionnaire fatigue or objectionable/unclear items).

*4. Adaptation*, the need to make intervention or procedural changes, was assessed through the qualitative interviews conducted post-intervention.

*5. Limited efficacy testing*, considered whether the intervention led to changes in depression symptoms at the mid-point and post-intervention. Limited efficacy was also assessed for secondary outcomes, including adolescent nutrition attitudes and practices, parent food modelling and the family food environment.

### Data analysis

The analyses were descriptive and exploratory. Quantitative continuous data were reported as means and standard deviations; categorical data were reported as frequencies and proportions. Qualitative data obtained through audio-recorded, semi-structured interviews were transcribed verbatim, de-identified and subject to inductive thematic analysis using NVivo12 software^(62,63)^. The thematic analysis procedure followed the guidelines outlined by Braun and Clarke (2006)^(64)^. Initially, each member of the research team conducted an independent review of the transcripts to immerse themselves in the data and gain a comprehensive understanding. Subsequently, individual team members created initial codes, which were later collectively compared and synthesised to form overarching themes through collaborative discussions. Upon achieving consensus on the most representative themes and subthemes, excerpts were selected from the transcripts to support and illustrate each theme.

Limited efficacy testing was conducted through reported means, standard deviations (sd), effect sizes (i.e. Cohen’s *d*) and 95 % CI. As this current study was not powered for hypothesis testing, effect sizes are reported to communicate the size and the direction of the potential treatment effect^(65)^. We used the guidelines by Cohen to interpret the magnitude of the effect sizes, namely small (0·2), moderate (0·5) and large (0·8)^(66)^. All quantitative analyses were performed using R statistical software^(67)^.

## Results

**Fig. 2.**
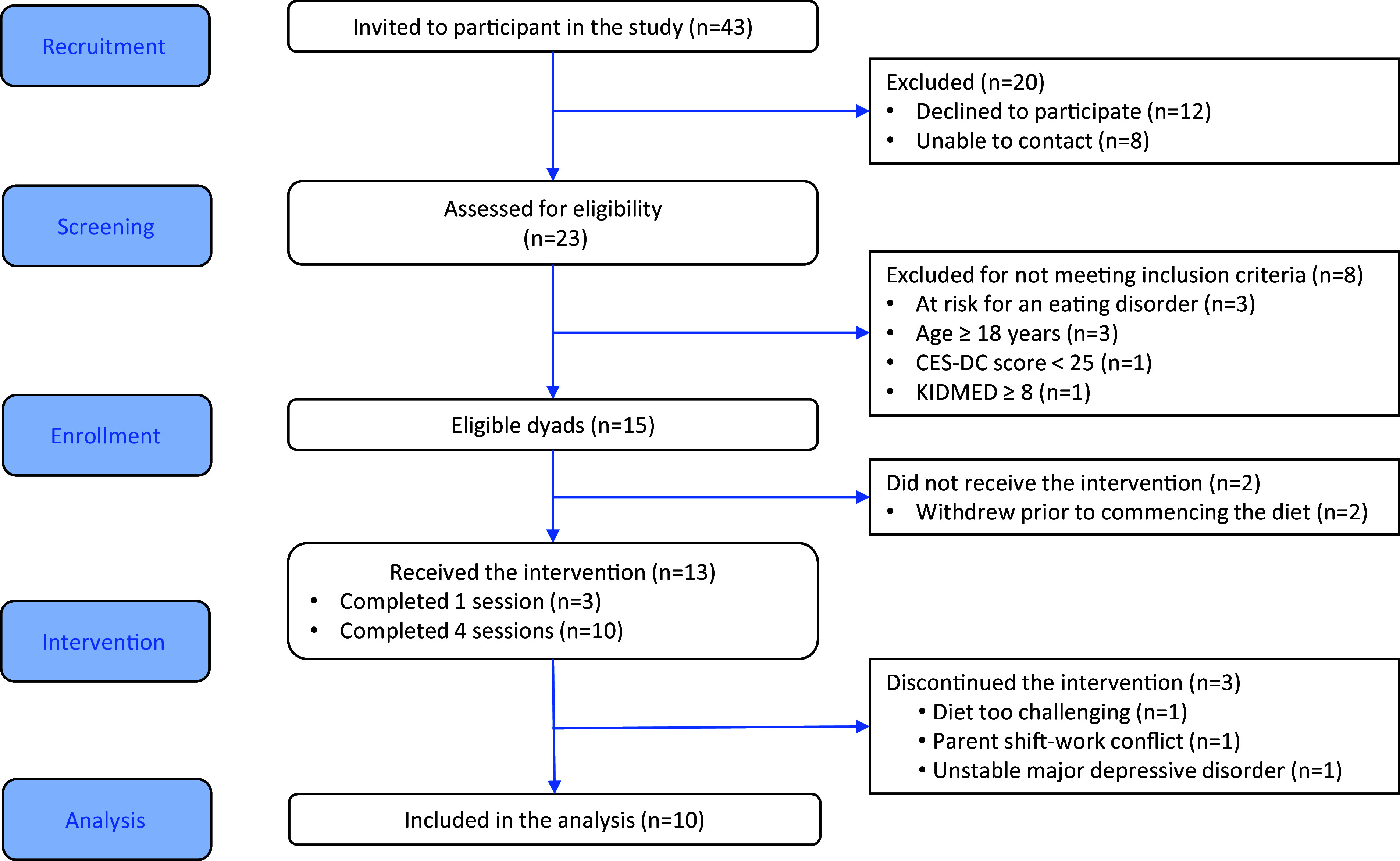
CONSORT participant flow diagram.

Forty-three adolescent–parent dyads were approached to participate in the study, twenty were not interested or did not respond to the invitation and eight did not meet the eligibility criteria. Fifteen dyads consented to participate in the study. Two dyads withdrew prior to commencing the study, and three dyads withdrew after completing one session: one found the diet too challenging and two withdrew for unrelated reasons (Fig. 2). Participating adolescents (*n* 10) had a mean age of 15·3 (sd = 1·4) years and were 100 % female. Participant characteristics are presented in Table 1. The study was well-tolerated with no adverse events reported.

**Table 1.** Participant characteristics (Mean values and standard deviations; Numbers and percentages)

Characteristic	Mean	sd
Adolescent (*n* 10)		
Age (years)	15·3	1·4
Sex (% female)	10	100
Anthropometry		
BMI (pre-intervention)	21·3	2·7
Race/ethnicity		
White/Caucasian	5	50
Mixed	2	20
South Asian/Asian	2	20
Other	1	10
	***n***	**%**
Gender		
Cis female	7	70 %
Did not respond	3	30 %
Sexual orientation		
Heterosexual	3	30 %
Non-heterosexual	3	30 %
Did not respond	4	40 %
Living situation		
Two parent household	5	50 %
Single parent household	5	50 %
Parent (*n* 10)		
Relationship to youth		
Mothers	7	70 %
Fathers	3	30 %
Household income		
< CAD 75 000/year	3	30 %
> CAD 75 000/year	3	30 %
Prefer not to answer	4	40 %

CAD, Canadian Dollar.

### Feasibility outcomes

#### Demand

Of the forty-three adolescent–parent dyads that were approached to be part of the study, fifteen consented to be part of the study, resulting in a recruitment rate of 40 % (see flow diagram, Fig. 2). Pre-intervention interviews offered further insight into reasons for participating in the study and anticipated barriers to study participation (Table 2).

**Table 2.** Pre-Intervention qualitative interview themes, mentions and illustrative quotes, *n* 10

Theme/Subtheme	Number of times mentioned		Illustrative quote
	Adolescent	Parent	
1. Reasons for study enrollment			
1.1. Family benefit	2	3	*‘So being that this is kind of a family study, I think that’s probably a good thing. Because if it was, you know, we are sending you food that you have to make for yourself as a teenager, uh, I don’t know if that would be successful. But as far as it being a family thing, maybe we’ll cook together, maybe, you know, we’ll learn together. […] So this is a, a good learning opportunity for both of us.’ - Parent_05*
1.2. Desire to modify eating behaviours	4	1	*‘Hopefully, I’ll be able to, like, discover more types of food and more types of cooking and stuff. So, I’ll be able to maybe like, […]be more motivated to incorporate that in my diet afterwards as well […] and incorporate it into my life in college next year’ - Adolescent_05*
1.3. Desperate and willing to embrace new depression management strategies	1	3	*‘Of course, the goal is to let her feel better, right? It has been a struggle. So, like I mentioned, anything we are willing to try anything that would make her feel better.’ - Parent_12*
1.4. Build adolescent agency for healthy eating habits	–	4	*‘Sometimes if I don’t feed her, she doesn’t eat so it’d be nice for her to take control of this, benefiting her and herself, and wanting to do it for herself.’ - Parent_04*
1.5. Contribute to research	3	1	*‘I just want to be able to help in any way that I can and find any sort of solution to any problem that somebody might have.’ - Adolescent_12*
1.6. Learn about nutrition	3	–	*‘I’m gonna help cook it, I want to do that because I’m, I have a food and nutrition class. And I’m learning to cook, and I want to be able to learn to cook. And I want to be able to customize the ingredients that you guys give me into ways that I like it. And try and find different ways that I can cook myself health- healthier meals.’ - Adolescent_12*
2. Anticipated barriers to intervention adherence			
2.1. Implementing diet into current lifestyle	4	5	*‘I think like all teenagers, she’s a little bit lazy when it comes to food. And so on the one hand, if I make it, she’ll eat it. But on the other hand, you know, she would just as easily, you know, either eat nothing or eat, you know, unhealthy choices, either because it’s easier, or because she’s craving something, or maybe she’s with friends or something like that.’- Parent_05*
2.2. Food preferences	2	–	*‘I’m really nervous honestly, about uh making changes to my diet just because like I’m kind of embarrassed about how, like, how limited my diet is. Just because I don’t know, I’m kind of a picky eater. And I just, I don’t know, I- I’m nervous for that […] the consistency too, um, to keep eating, like, on the I know, is not like an official plan, but it’s still it’s still going to be a change, and it’s still going to have to be one that I’m gonna have to get used to. And I’m gonna have to actually, like, take some time, and like reinforce’ - Adolescent_11*

Motivations for study participation were categorised into six subcategories (labelled 1·1–1·6). Firstly (1·1), parents (*n* 3) and adolescents (*n* 2) highlighted the perceived benefits for the entire family, emphasising the belief that involvement in the study would bring positive changes not only to adolescents but also to the well-being of the family as a whole. Secondly (1·2), parents (*n* 1) and adolescents (*n* 4) expressed hope for potential improvements in their adolescents’ eating habits and considered study participation as a means to positively influence adolescents’ dietary choices. Thirdly, parents (*n* 3) and adolescents (*n* 1) reported that another motivating factor for study participation was their desperation to improve depression symptoms (1·3). Parents (*n* 4) also expressed a strong desire for adolescents to become more self-aware of their eating habits (1·4) and saw the study as an opportunity to foster greater self-awareness and healthier eating choices. Some adolescents (*n* 3) and parents (*n* 1) reported more altruistic motives, expressing a desire for their participation to benefit not only themselves but also others who might be in a similar situation in the future (1·5). Lastly, adolescents (*n* 3) expressed genuine interest in learning about nutrition (1·6), highlighting curiosity and eagerness to expand their knowledge in this area.

Participants also identified anticipated barriers to intervention adherence, which were categorised into two subcategories (labelled 2.1 and 2.2). The first subcategory (2·1), anticipated barriers to intervention adherence, encompassed a range of potential practical difficulties. Parents (*n* 5) and adolescents (*n* 4) were concerned about managing fluctuations in the adolescent’s mood, potential distractions from friends, deviation from current eating habits, intervention duration and potential scheduling conflicts with family activities such as summer holidays during the summer months, or extracurricular engagements during the school year. The second subcategory, dietary preferences (2·2), centred only on adolescents’ concerns (*n* 2) that they may not like the food that comprised the nutrition intervention, due to their self-professed pickiness as eaters.

#### Acceptability

Acceptability was determined using the AIM and qualitative interviews. All adolescents and their parents completed the AIM questionnaires pre- and post-intervention. The AIM remained relatively consistent for both parents and adolescents pre- and post-intervention (AIM parent mean pre-intervention scores 17·90 (sd = 2·08) *v*. post-intervention scores 17·90 (sd = 1·91); AIM adolescent mean pre-intervention scores 15·78 (sd = 1·92) *v*. post-intervention scores 16·0 (sd = 2·11)

**Fig. 3.**
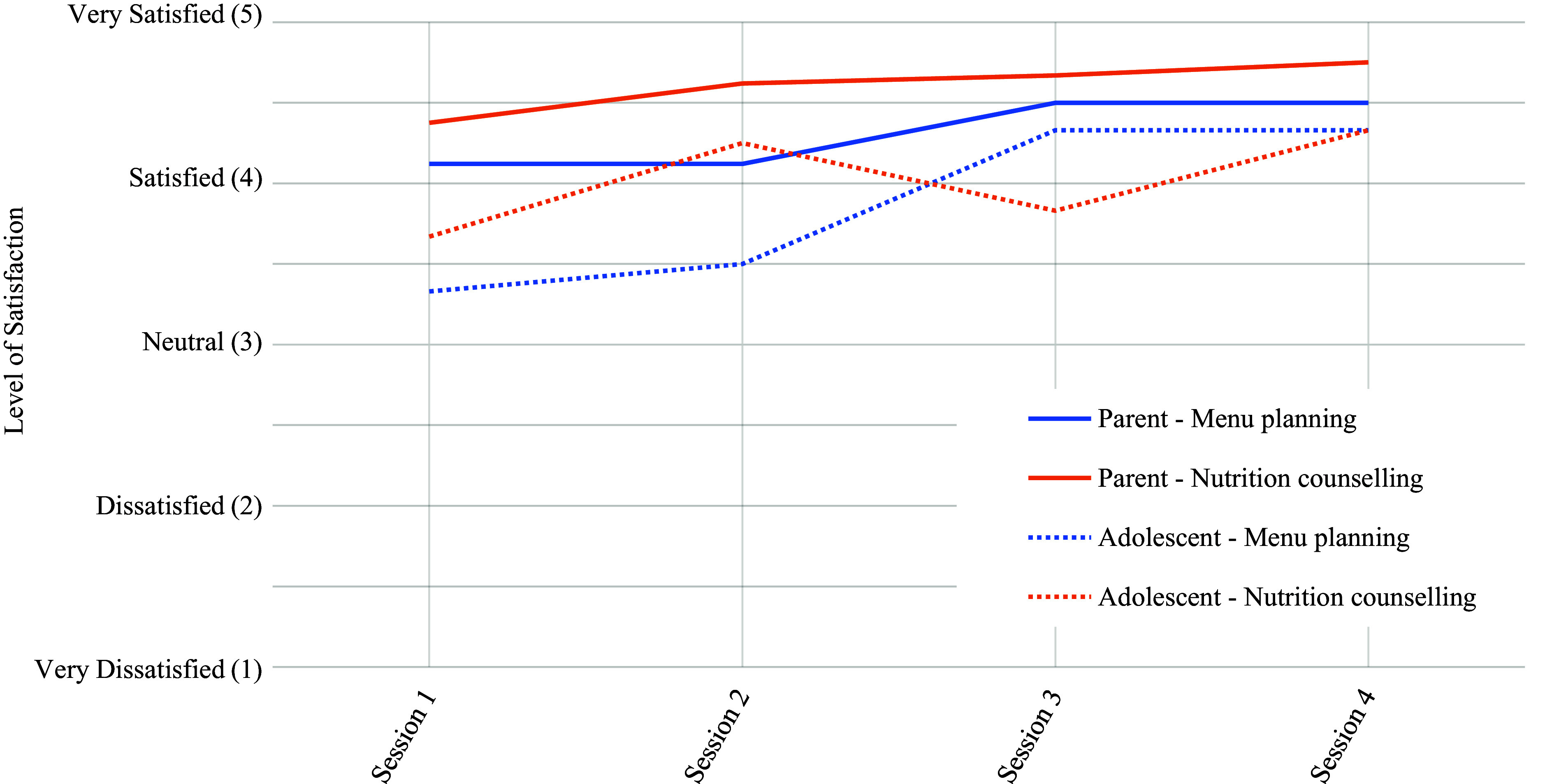
Satisfaction scores for menu planning and nutrition counselling for adolescents and parents, by session (*n* 10).

Parents expressed a high level of satisfaction with menu planning and nutrition counselling, while adolescents demonstrated an adequate level of satisfaction with menu planning and nutrition counselling (Fig. 3).

Post-intervention interviews were coded into three categories to facilitate the interpretation of acceptability data: (1) perceived benefits of study participation; (2) factors contributing to study satisfaction and (3) challenges to study participation. Table 3 summarises interview categories and representative quotes.

**Table 3.** Post-Intervention qualitative interview theme, mentions and illustrative quotes, *n* 10

Theme/Subtheme	Number of times mentioned		Illustrative quote
	Adolescent	Parent	
1. Benefits of study participation			
1.1. Opportunity to try new foods	4	4	*‘It was a good experience because I got to choose like a variety of different things that I haven’t really tried before. […] I was introduced to, like, new stuff. It kind of opened my eyes on like different things I could use in my meals in the future that I didn’t really think of’- Adolescent_015*
1.2. Continue with new eating habits post-intervention	4	5	*‘I definitely want to eat more healthy. So, I’ll probably keep up; like one of my goals was eating four vegetables a day. So probably like keep up with that goal.’ - Adolescent_008*
1.3. Involve family members in the process	1	1	*‘My younger child had a positive experience as well because she is also affected by it because when I cook, she also gets to eat right. […] It brought about good eating habits for her [younger daughter]. […] So, it not only impacted the participant but the family member as well.’- Parent_002*
2. Factors influencing study satisfaction			
2.1. The program’s flexible pace	4	4	*‘Because, you know, [nutritionist’s name] […] was very flexible with, with uh what we could do and just concentrate on the small changes.’ - Parent_011*
2.2. Finding healthy food choices	3	3	*‘It’s just um the ability to like substitute some of the foods or some of the recipes that weren’t working, and also just trying to make like, small changes to uh my diet already. […] And like, even when, like, stuff wasn’t working like the nutritionist, you know, like, she was very quick to think of alternatives and um just things that uh things that would be good to change […] I found out new grains and healthier alternatives’ - Adolescent_011*
2.3. Menu planning software	4	1	*‘Having the website that we used was really motivating. Because it, it laid out all of my options and stuff for me. And it was easy to sift through easy to look through. And like it, it helps like when you have things like written up like that it’s way easier, and it motivates you to get more like, changes and get stuff done.’ - Adolescent_012*
2.4. Virtual counselling sessions	4	4	*‘Well, the virtual is definitely convenient. So, it’s not really a hands-on thing right. Like, like the the person doesn’t need to, …, you don’t need to physically do anything. So, then I think that virtual is, is probably okay. For this, I mean, it definitely saves time and driving and parking and, you know, time and that kind of thing they more people are available to do it when it’s convenient like that, right?’- Parent_005*
2.5. Grocery delivery	2	*3*	*‘And it was also good because like, once you have that food, there’s really no excuse to, you know, have a go to waste. […] So, it was good to also have that [the deliveries] as an incentive to eat better and follow the planning’ - Adolescent_011* *‘And not having to think about it, too, was handy. Because that, you know, buying the ingredients is sometimes half the battle and having it available was really helpful, too. So it was all good.’ Parent_011*
3. Challenges to study participation			
3.1. Long meal preparation time	2	2	*‘[There were] definitely like difficult parts of it. […] everything kind of has to be made, like by scratch kinda, or like, everything has to be cooked, like, it’s not a fast food or anything type of diet. And so, because of that, like it kind of made I guess, preparation for things a bit longer.’ - Adolescent_010*
3.2. Timing of the intervention	1	1	*‘As we started this, and then school hit, it was really hard to do a lot of the things once we got into our hectic lifestyle, you know, […] just the practicality of trying to do these new things on a very hectic schedule with other kids and you know [participant’s name]'s brother having hockey and my work, you know, in the evenings and stuff. So that was our own challenges.’ - Parent_008* *‘Um, just because like at the summer, like in the summer at my cottage, it was just not the ideal sort of place doing it. […] I didn’t necessarily have time to like, prep food and stuff like’ - Adolescent_004*

In the first category, ‘Benefits to Study Participation,’ participants recognised several advantages (labelled 1·1–1·3). Within the first subcategory (1·1), parents (*n* 4) and adolescents (*n* 4) recognised that the study provided an opportunity for adolescents to try new foods. This allowed them not only to expand their dietary choices but also to incorporate new food into their eating habits (1·2). Parents (*n* 5) and adolescents (*n* 4) expressed the intention to continue eating new food they tried during the intervention emphasising the sustainability of these positive changes beyond the study period. Lastly, one parent and one adolescent mentioned that the study involved family members outside the dyads outside the recruited dyads (1·3).

Within the second category, ‘Factors Influencing Study Satisfaction’, five subcategories emerged, elucidating the factors contributing to study satisfaction (labelled 2.1–2.5). One key aspect highlighted was the flexibility of the intervention’s pace (2·1), as both parents (*n* 4) and adolescents (*n* 4) appreciated the ability to adapt the intervention to their individual goals and schedules. Another factor that emerged as contributing to adolescent satisfaction was the wide variety of acceptable foods that were available for them to select (2·2), which was mentioned by parents (*n* 3) and adolescents (*n* 3). This increased the feasibility of the intervention for adolescents in that they were able to meet their food goals while still enjoying their meals. Parents (*n* 1) and adolescents (*n* 4) also commended the menu planning software for its personalisation features, including grocery lists, menu plans and recipes (2·3). This feature not only simplified meal planning but also added a level of personalisation that resonated with participants, enhancing their overall experience. The virtual nature of the nutrition counselling sessions (2·4) was another factor positively influencing study satisfaction. Both parents (*n* 4) and adolescents (*n* 4) found virtual sessions convenient and appreciated the accessibility of nutrition counselling from the comfort of their homes. Lastly, grocery deliveries received positive feedback from adolescents (*n* 2) and parents (*n* 3) (2·5). This service streamlined the process of obtaining the novel ingredients for the intervention and allowed adolescents to explore new foods they may have not tried otherwise.

In the third category, ‘Challenges to Study Participation,’ two subcategories emerged and provided insights into the difficulties that participants faced during the study (labelled 3.1–3.2). Parents (*n* 2) and adolescents (*n* 2) thought that the time required for meal preparation was too lengthy (3·1). Parents (*n* 1) and adolescents (*n* 1) also raised implementation concerns about the intervention timing with respect to the time of year (3·2), suggesting that external factors could impact participation and adherence. Of note, however, participant perspectives regarding the optimal time of year in which to implement a new dietary intervention were discrepant, with some participants suggesting the stress-free summer time would be a better time, while others suggested that the more routinised academic year would allow for easier implementation.

#### Implementation

Implementation feasibility was determined using the FIM and by intervention adherence, participant attrition and completion rate of measures. Mean FIM scores for both parents and adolescents showed relative stability across pre- and post-intervention assessments. Parents’ scores averaged 15·80 (sd = 2·78) pre-intervention and increased slightly to 16·60 (sd = 2·84) post-intervention. Similarly, adolescents’ scores remained steady at 16·33 (sd = 2·06) pre-intervention and 16·00 (sd = 1·89) post-intervention. Over the 8-week intervention, adherence to the diet, as assessed by the KIDMED, demonstrated improvement with a large effect size between baseline and midpoint (Cohen’s *d* = 0·80, 95 % CI (0·24, 2·41)), and a moderate effect size between baseline and post-intervention (Cohen’s *d* = 0·64, 95 % CI (0·15, 1·33))). Of the thirteen dyads that initiated the study, ten completed the study for an attrition rate of 23 %. Reasons for withdrawing from the study included personal (non-intervention) reasons (*n* 2) and a challenging diet (*n* 1) (Fig. 2).

While all REDCap measures achieved 100 % completion across all three timepoints, dietary data collection using the ASA-24 had suboptimal completion rates. Six participants completed at least one ASA-24 recall at baseline and mid-point, including two incomplete baseline recalls with implausibly low daily caloric intake (less than 500 kcal). At the final time point, four participants completed a single ASA-24; one participant provided an incomplete recall with a similarly low caloric intake. The low completion rate prevented the assessment of diet quality using the HEI-2015. Completion of the anthropometric data was 90 % at the midpoint and 70 % at the end of the study time point.

#### Adaptation

Participant feedback from post-intervention interviews highlighted some procedures requiring adaptation. Participants also made several suggestions with respect to adaptation of the intervention for the study team to consider: adolescents (*n* 6) reported that they either did not receive or did not read the eHealth messages; one adolescent suggested making the menu planning software accessible to both the nutritionist and participants, to allow adolescents to explore recipes at their own pace; two parents expressed interest in having more frequent (weekly instead of biweekly sessions) to discuss study progress; three parents expressed interest in receiving more information about the principles of the Mediterranean Diet during the initial nutrition counselling session and two parents recommended providing paper copies of menu plans and recipes for their convenience.

#### Limited efficacy testing

Effect sizes for adolescent and parent-reported outcome scores are presented in Table 4. Small effects on depressive symptoms were observed (Cohen’s *d* = 0·36, 95 % CI (–0·24, 3·36)). We also observed improvements in parent food modelling (Cohen’s *d* = 0·24, 95 % CI (–0·43, 1·16)), adolescent nutrition attitudes (Cohen’s *d* = 0·36, 95 % CI (–0·25, 1·33)) and substantial enhancements in the family food environment (Cohen’s *d* = 0. 61. 95 % CI (–0·04, 2·61)). Adolescents’ nutrition knowledge was average at 74 % (14·8/20) pre-intervention and 78 % (15·5/20) post-intervention, corresponding to a negligible increase (Cohen’s *d* = 0·11, 95 % CI (–1·10, 0·53)).

**Table 4.** Effect size (Cohen’s *d*) for outcome measures at pre-intervention, mid-point and, post-intervention, *n* 10 (standard deviations and 95 % CI)

Measure	Pre-intervention	sd	Mid-point	sd	Post-intervention	sd	Cohen’s *d*	95 % CI
CES-DC	33·67	10·91	30·67·	8·23	29·1	7·20	0·18	–0·49, 1·11^¶^
							0·36	–0·24, 3·36^†^
PMQ	14·11	7·52	NA		15·60	5·42	0·24	–0·43, 1·16
FNPA	46·2	5·63	NA		50·4	3·92	0·61	–0·04, 2·61
Nutrition knowledge	14·8	5·43	NA		15·50	3·03	0·11	–1·10, 0·53
Nutrition attitudes	17·33	2·00	NA		18·00	1·70	0·36	–0·25, 1·33

CES-DC, Center for Epidemiological Studies Depression Scale for Children; PMQ, Parent Modelling Questionnaire; FNPA, family and nutrition and physical activity; NA, not assessed. ^¶^pre-intervention *v*. midpoint; ^†^pre-intervention *v*. post-intervention.

## Discussion

The current study examined the feasibility of implementing a novel 8-week personalised nutrition intervention among adolescents with MDD. We assessed the feasibility of this intervention across various dimensions, encompassing demand, acceptability, implementation and a preliminary exploration of its efficacy.

In this study focusing on a new intervention for adolescents with MDD and their parents, we achieved a recruitment rate of 43 %. This is similar to a dietary intervention study for young adults (mean age of 19·6 years) with depression (40 %)^(68)^ and a trial of a dietary intervention for adults with MDD (42 %)^(69)^. Among studies of adults, younger age, male gender and lower socio-economic status have been associated with decreased research participation^(70,71)^. However, fewer studies have examined factors affecting the recruitment of children and adolescents for dietary intervention studies, and we are not aware of any research that has examined correlates of research participation of adolescent and parent dyads in this field. Among the broader literature regarding child and adolescent research recruitment, no significant associations were observed between age or gender; however, higher parental educational level has been associated with higher enrollment rates^(72)^. Further investigation into factors affecting recruitment, particularly for dietary inventions, is warranted.

The pre-intervention interviews provided insight regarding motivations and barriers to study participation and offered potential areas for adjustment to the recruitment strategy. Parents’ reasons for study participation aligned with some adolescents’ reasons but not always to the same degree. For example, both parents and adolescents were hopeful that the intervention would improve depression symptoms, with parents also sharing their own feelings of desperation with respect to finding an effective treatment for their child’s depression. More adolescents than parents expressed altruistic motivations for participating in the current study, which aligns with adolescent motivation in similar research^(73)^. Both parents and adolescents foresaw challenges when it came to incorporating a higher-quality diet into their existing lifestyles. These challenges included practical obstacles, such as the perceived time and effort required, as well as managing mood fluctuations related to adolescent depression symptoms. Additionally, maintaining adherence to the personalised nutrition intervention was seen as a potential hurdle, influenced by individual food preferences and dislikes. Finally, juggling busy schedules, including school, extracurricular activities and holiday plans, posed yet another obstacle to successful diet integration. However, only adolescents mentioned selective food preferences as a barrier to intervention adherence. Common reasons for declining research participation in the current study (e.g. parent was too busy, might dislike included foods) also align with those of previous research^(74)^. This suggests that addressing potential barriers to recruitment proactively, by highlighting the personalised and adaptable nature of the dietary intervention, will be important in future research to improve recruitment rates.

Convergence of quantitative and qualitative data including responses to validated questionnaires and themes generated from parent and adolescent interviews in the current study suggests that a personalised nutrition intervention is acceptable among adolescents with depression and their parents. The high level of intervention acceptability and satisfaction among adolescents and parents indicates that the programme was generally well-received. Furthermore, satisfaction with menu planning and nutrition counselling was generally high, with parents reporting slightly higher satisfaction than adolescents. The attrition rate of 23 % indicates moderate success in retaining participants throughout the intervention and is similar to other adolescent lifestyle interventions (i.e. general nutrition and/or exercise) for depression (15–35 %)^(75–77)^. Post-intervention interviews revealed that participants recognised several benefits of study participation, such as trying new foods, expanding dietary choices and involving family members in the process. Other factors contributing to the acceptability and satisfaction with the intervention included the flexibility of the dietary components, the availability of acceptable food choices, personalised menu planning software, virtual nutrition counselling and the convenience of grocery deliveries.

The ASA-24, a self-reported dietary intake measure, had the lowest completion rates among the study measures, and many reports contained missing data. To address this issue, more support may be required when completing this measure in future trials. For example, ASA-24 assessments could be conducted during nutrition counselling sessions, or alternate dietary measures could be incorporated to collect self-report data via REDCap or employ novel, specialised, food photography apps to document participants’ dietary intake.

This study offers preliminary evidence for the effectiveness of the intervention in reducing depressive symptoms among adolescents with MDD. Additionally, the improvement in diet quality (KIDMED score) suggests a positive impact on dietary habits. These preliminary results are consistent with the only other study that has conducted a dietary intervention aimed at reducing depression symptoms among young people with depression, which reported lower self-reported depression symptoms using the Center for Epidemiologic Studies Depression Scale – Revised and high dietary compliance^(68)^. Furthermore, the current study provides early evidence for improvements in parent food modelling, adolescent nutrition attitudes and the family food environment. The negligible change in adolescent nutrition knowledge may be attributed to the already reasonably high levels of participants’ nutrition knowledge at baseline and the lack of formal nutrition education during counselling sessions, which may have limited the potential for significant improvement.

### Limitations

The interpretation of the efficacy of the intervention should be considered with caution, as the focus of this study was on feasibility. Moreover, the absence of a control group makes it challenging to determine whether the observed positive impacts can be attributed solely to the intervention or if other variables (i.e. the non-specific benefits of meeting regularly with an interested health professional) also contributed to the improvements in depressive symptoms observed. Additionally, as the study period was reasonably brief (8 weeks), whether the dietary and depression changes found in the current study will be sustained in the longer term is not known^(78–80)^. Data regarding the effect of the intervention on dietary intake should be interpreted with caution, given both the small initial sample size and the incomplete data, as three participants did not complete the intervention. It is also possible that social desirability bias may have affected self-reported dietary intake and health outcomes; however, adolescents with MDD have been shown to report their energy intake at levels comparable to that of other adolescents, indicating similar potential effects of social desirability bias on dietary intake reporting irrespective of MDD diagnosis^(81–83)^. The potential effect of medication on appetite was not explicitly explored in this pilot study. Future trials with larger sample sizes should address this limitation by including an examination of the intervention’s potential effects on underlying appetite trends. This examination should consider the potential influence of disease course and medications used in clinical care. Finally, FIM and AIM scores reflect data from participants who completed the entire study. Three participants who withdrew after the first session are therefore excluded from the presented FIM and AIM results.

### Conclusions

This study found that a newly developed personalised nutrition intervention was feasible according to the criteria outlined by Bowen *et al.* ^(62)^ for adolescents with MDD and their families. Future studies should consider recruitment strategies that mitigate hesitation about dietary change by proactively highlighting the flexible and customisable nature of the personalised nutrition intervention that accommodates individual food preferences. These insights lay the groundwork for future research examining the potential effectiveness of family-based, personalised nutrition interventions in the treatment of adolescent depression.
